# LASSO-based nomogram and machine learning models for predicting 30-day nasogastric tube dependence after acute ischemic stroke

**DOI:** 10.3389/fneur.2026.1811560

**Published:** 2026-08-10

**Authors:** Qianqian Shang, Yu Lei, Hongguang Chen, Zhongli Liu, Minyue Sun, Shuang Wang, Shiyu Wen, Yingying Deng, Min Luo

**Affiliations:** Department of Neurology, Mianyang Central Hospital, School of Medicine, University of Electronic Science and Technology of China, Mianyang, Sichuan, China

**Keywords:** acute ischemic stroke, LASSO regression, machine learning, nasogastric tube, nomogram

## Abstract

**Background:**

Nasogastric tube (NGT) feeding is a common method for providing enteral nutrition to patients with acute ischemic stroke (AIS) and dysphagia. However, prolonged NGT dependence contributes to adverse clinical consequences. Early identification of patients who may develop persistent NGT dependence remains challenging.

**Methods:**

A total of 852 AIS patients requiring NGT were included and allocated to the training (*n* = 596) and internal validation (*n* = 256) sets. Predictor selection utilized least absolute shrinkage and selection operator (LASSO) regression, the eXtreme Gradient Boosting (XGBoost) algorithm, and multivariate logistic regression. A nomogram was then constructed and internally and externally validated. Three machine learning algorithms-Gradient Boosting Machine (GBM), Support Vector Machine (SVM), and Regularized Discriminant Analysis (RDA) were also developed for comparison. Model performance was evaluated using the Area Under the Curve (AUC), calibration plots, and Decision Curve Analysis (DCA).

**Results:**

Four key independent predictors were retained: Diabetes Mellitus (DM), age, admission National Institutes of Health Stroke Scale (NIHSS) score, and exclusive NGT feeding. The model demonstrated strong discrimination (AUC = 0.924 in training, 0.920 in internal validation, and 0.935 in external validation), good calibration, and favorable clinical utility in DCA. Among the machine learning models, GBM demonstrated the highest accuracy, with an AUC of 0.918. The model confirmed age and NIHSS score as the most influential predictors, followed by exclusive NGT feeding and DM.

**Conclusion:**

The developed nomogram provides an effective approach for predicting 30-day NGT dependence in AIS patients, enabling timely risk stratification and individualized clinical management.

## 1 Introduction

Dysphagia frequently occurs as a neurological complication after stroke, arising from cortical and brainstem injury that disrupts swallowing reflexes and airway coordination, with an incidence of 34–80% in patients following acute ischemic stroke (AIS) (1, 2). Post-stroke dysphagia not only compromises the nutritional intake and leads to malnutrition, but can also cause dehydration, aspiration pneumonia, and airway obstruction, ultimately contributing to poor prognosis and mortality (3–6). While many patients experience gradual recovery of swallowing function within a few weeks after stroke, a considerable proportion remain dependent on enteral tube feeding beyond the acute phase, either through a nasogastric or gastrostomy tube (7, 8). However, in East Asia, gastrostomy is not widely accepted, and most stroke survivors with dysphagia receive nasogastric tube (NGT) feeding during the acute and rehabilitation phases (9, 10).

NGT feeding is a convenient and non-invasive method of enteral nutrition support, widely used for sustaining nutrition and hydration in patients with impaired swallowing function after stroke (11, 12). NGT feeding is generally appropriate for short-term nutrition support, typically lasting no more than 4 weeks (13). Prolonged utilization of NGT is linked to multiple complications, such as nasal alar erosion, chronic sinusitis, aspiration pneumonia, upper gastrointestinal bleeding, and gastroesophageal reflux (10, 14, 15). Research has further demonstrated that presence of an NGT can disrupt normal epiglottic movement during the pharyngeal phase of swallowing (16). These complications can adversely impact the prognosis of stroke patients, while timely NGT removal has been shown to lower mortality rates (17). Consequently, early identification of individuals prone to prolonged NGT dependence is crucial for tailoring swallowing rehabilitation and improving post-stroke recovery.

Previous studies have reported that patients with higher National Institutes of Health Stroke Scale (NIHSS) scores, advanced age, dysarthria, aspiration during swallowing assessments, and prior intubation are at increased risk of prolonged dysphagia lasting beyond six weeks (18–20). Several efforts have been made to establish predictive models for NGT removal and swallowing recovery after stroke, incorporating variables including age, body mass index (BMI), admission stroke severity, lesion location, aspiration risk, functional oral intake, activities of daily living, and NIHSS score (7, 21–23). However, most of these models were designed by traditional regression-based approaches, and their predictive precision and generalizability remain limited. The nomogram and machine learning models can enhance prediction by handling complex, high-dimensional data (24, 25).

The present study focused on constructing and validating a LASSO-based nomogram and machine learning models to predict 30-day NGT dependence in patients after AIS, supporting individualized clinical decisions and early intervention planning.

## 2 Methods

### 2.1 Study selection

Overall, 852 patients diagnosed with AIS were retrospectively enrolled from the Department of Neurology, Mianyang Central Hospital between January 1, 2024, and May 31, 2025. AIS diagnosis was established using brain computed tomography (CT) or magnetic resonance imaging (MRI) findings, in accordance with World Health Organization criteria. Patients were included if they (1) clinically confirmed dysphagia after stroke, (2) completed the 30-day follow-up evaluation, (3) placement of a NGT for enteral feeding during hospitalization, and (4) were ≥ 18 years of age. Exclusion criteria were: (1) incomplete clinical records, (2) history of swallowing disorders or neurological diseases affecting swallowing function, (3) concurrent intracerebral hemorrhage or subarachnoid hemorrhage, or (4) death within 30 days after stroke onset.

Eligible patients were assigned to a training cohort (*n* = 596) and an internal validation cohort (*n* = 256) in an approximate 7:3 ratio to facilitate model development and validation. Baseline demographic and clinical characteristics showed no statistically significant differences between the two groups.

To further evaluate the robustness and generalizability of the predictive model, an external validation cohort was obtained from Mianyang Central Hospital, comprising patients admitted between January 1, 2023, and December 31, 2023. This cohort included 344 patients with AIS who met the same eligibility criteria. Baseline demographic and clinical profiles of the external validation cohort were comparable to those in the internal dataset.

### 2.2 Definition of nasogastric tube dependence

NGT dependence was defined according to the WST score assessed 30 days after AIS. A WST score ≥ 3 was considered indicative of NGT dependence, as this threshold reflects impaired swallowing reflexes, manifested by coughing, choking, or hoarseness during water swallowing, suggesting a high risk of aspiration. In contrast, a WST score < 3 were classified as the non-NGT-dependent group, reflecting relatively preserved swallowing function (26). Among 852 eligible patients, 367 patients experienced persistent 30-day NGT dependence, with 257 patients in the training set and 110 patients in the internal validation set.

### 2.3 Data collection

Demographic, clinical, and laboratory data at baseline were obtained from the hospital information system. Variables included: sex, age, BMI, high blood pressure (HBP), diabetes mellitus (DM), hyperlipidemia (HPL), coronary heart disease (CHD), atrial fibrillation (AF), chronic cardiovascular disease (CVD), smoking and drinking history, previous stroke (number of previous stroke times), hemorrhagic transformation (HT), National Institutes of Health Stroke Scale (NIHSS) score on admission, aspiration pneumonia, exclusive NGT feeding, serum C-reactive protein (CRP), serum albumin, and infarction location.

Infarction location was classified according to the vascular territory of ischemic stroke as anterior circulation infarction (coded as 1), posterior circulation infarction (coded as 2), or combined anterior and posterior circulation infarction (coded as 3).

Exclusive NGT feeding was recorded as a baseline binary variable at admission. Patients receiving all nutritional intake via a NGT were classified as having exclusive NGT feeding (coded as 1), whereas those who were able to tolerate partial oral intake in combination with NGT feeding were classified as partial NGT feeding (coded as 0).

### 2.4 Variable selection and nomogram development

To reduce overfitting and select the most predictive variables, least absolute shrinkage and selection operator (LASSO) regression with 10-fold cross-validation was performed on the training cohort to identify the optimal penalty parameter (*λ*). Predictors retaining non-zero coefficients under the chosen *λ* were then entered into subsequent analyses and nomogram construction. The process and tuning parameter (λ) were visualized in the cross-validation curve and coefficient path plot.

The eXtreme Gradient Boosting (XGBoost) is a gradient boosting ensemble method capable of handling nonlinear interactions and multicollinearity. It was implemented to assess the relative importance of each variable. Variable importance was quantified using feature contribution scores (gain), and the top-ranking predictors were visualized in a feature importance plot.

Univariate logistic regression was initially performed to identify factors associated with 30-day NGT dependence. Factors with statistical significance (*p* < 0.05) in the univariate analysis were then entered into a multivariable logistic regression model to determine independent risk factors. Odds ratios (ORs) with 95% confidence intervals (CIs) were reported. The final set of predictors was derived from the overlapping variables identified by combining independent predictors retained through multivariate logistic regression, features chosen through LASSO regression, and those ranked highly in the XGBoost feature importance analysis. A nomogram was developed to provide individualized estimates for the probability of 30-day NGT dependence.

In the nomogram, the value of each predictor corresponds to a specific point on the uppermost scale. The points assigned to all predictors are summed to obtain a total score, which is then projected onto the probability scale to estimate an individual’s risk of 30-day NGT dependence (27, 28). In addition to the static nomogram, the dynamic nomogram enables automatic calculation of the predicted risk after entering the corresponding predictor values.

### 2.5 Evaluation of nomogram performance

Nomogram performance was evaluated across the training, internal validation, and external validation cohorts. Discriminative ability was measured by the receiver operating characteristic (ROC) curve and the area under the curve (AUC). To determine calibration, we applied the Hosmer–Lemeshow goodness-of-fit test and generated calibration plots to compare predicted and actual results. Clinical usefulness was determined through decision curve analysis (DCA), which calculated the net benefit across various threshold probabilities.

### 2.6 Machine learning model construction and comparison

To further evaluate model performance, three machine learning algorithms—Gradient Boosting Machine (GBM), Support Vector Machine (SVM), and Regularized Discriminant Analysis (RDA)-were developed using the predictors of nomogram.

Each model’s performance was evaluated using five repeated resampling iterations to ensure robustness and reduce the impact of random data partitioning. In each iteration, key performance metrics, including AUC, precision, recall, and F1-score, were calculated.

To visually assess discriminative ability, ROC curves were plotted for the GBM model in both the training and internal validation sets. In addition, a lollipop plot was generated to illustrate the relative importance of the predictors in the GBM model.

### 2.7 Statistical analysis

Normally distributed continuous data are expressed as mean ± standard deviation (SD) and compared using the independent-samples t-test. Non-normally distributed continuous variables were reported as median (interquartile range, IQR) and compared with the Mann–Whitney U test. Categorical variables were expressed as frequencies (percentages) and analyzed using Fisher’s exact test or the chi-square test, depending on the data.

All statistical analyses were conducted in R software (version 4.2.2), and LASSO regression was carried out with the “glmnet” package. Nomograms and calibration curves were created using the “rms” package. Receiver operating characteristic (ROC) curves and decision curve analysis (DCA) were generated with the “pROC” and “rmda” packages, respectively. Machine learning models were built using the “caret” and “mlr” packages. A two-sided *p*-value < 0.05 was considered statistically significant.

## 3 Results

### 3.1 Characteristics of patients

Following application of the inclusion and exclusion criteria, a total of 852 patients with AIS were enrolled. They were randomly split into a training cohort (n = 596) and an internal validation cohort (*n* = 256). NGT dependence, defined according to the 30-day WST score (≥ 3 indicating dependence), was used as the primary outcome. The baseline characteristics of the training, internal validation, and external validation cohorts are summarized in Table 1. The mean age of the overall population was 67.73 years in the training set, 68.51 in the internal validation set, and 55.95 in the external validation set, with males accounted for 59.4, 57.0, and 57.3%, respectively. No statistically significant differences were found in any baseline variable between the training and internal validation groups (all *p* > 0.05), indicating good data comparability.

**Table 1:** Baseline characteristics of patients with acute ischemic stroke (AIS) in training and testing set.

Characters	Level	Training set	Internal validation set	*p*^	External validation set
*n*		596	256		344
Sex (%)	0	242 (40.6)	110 (43.0)	0.571	147 (42.7)
	1	354 (59.4)	146 (57.0)		197 (57.3)
HBP (%)	0	301 (50.5)	123 (48.0)	0.56	237 (68.9)
	1	295 (49.5)	133 (52.0)		107 (31.1)
DM (%)	0	432 (72.5)	178 (69.5)	0.428	307 (89.2)
	1	164 (27.5)	78 (30.5)		37 (10.8)
HPL (%)	0	448 (75.2)	191 (74.6)	0.931	298 (86.6)
	1	148 (24.8)	65 (25.4)		46 (13.4)
CHD (%)	0	476 (79.9)	214 (83.6)	0.24	272 (79.1)
	1	120 (20.1)	42 (16.4)		72 (20.9)
AF (%)	0	512 (85.9)	216 (84.4)	0.635	322 (93.6)
	1	84 (14.1)	40 (15.6)		22 (6.4)
CVD (%)	0	503 (84.4)	220 (85.9)	0.637	313 (91.0)
	1	93 (15.6)	36 (14.1)		31 (9.0)
Smoke (%)	0	403 (67.6)	183 (71.5)	0.300	241 (70.1)
	1	193 (32.4)	73 (28.5)		103 (29.9)
Drink (%)	0	508 (85.2)	216 (84.4)	0.828	296 (86.0)
	1	88 (14.8)	40 (15.6)		48 (14.0)
Previous stroke (%)	0	523 (87.8)	225 (87.9)	0.991	303 (88.1)
	1	42 (7.0)	17 (6.6)		23 (6.7)
	2	23 (3.9)	10 (3.9)		14 (4.1)
	3	8 (1.3)	4 (1.6)		4 (1.2)
HT (%)	0	363 (60.9)	209 (81.6)	0.405	309 (89.8)
	1	233 (39.1)	47 (18.4)		35 (10.2)
Infarction location (%)	1	439 (73.7)	196 (76.6)	0.650	N/A
	2	148 (24.8)	56 (21.9)		
	3	9 (1.5)	4 (1.6)		
Age (mean (SD))		67.73 (10.83)	68.51 (10.21)	0.325	55.95 (10.49)
BMI (mean (SD))		23.84 (2.97)	23.66 (2.81)	0.414	23.78 (3.03)
NIHSS score (mean (SD))		11.13 (6.23)	11.42 (5.92)	0.534	9.74 (5.44)
Aspiration pneumonia (mean (SD))		0.32 (0.47)	0.31 (0.46)	0.933	0.26 (0.44)
Exclusive NGT feeding (mean (SD))		0.45 (0.50)	0.41 (0.49)	0.267	0.35 (0.48)
CRP (mean (SD))		17.02 (17.85)	17.51 (18.91)	0.718	17.57 (18.61)
Albumin (mean (SD))		40.27 (3.36)	40.40 (3.48)	0.594	40.23 (3.42)

Unless otherwise specified, 0 represents “No” and 1 represents “Yes.” Variable definitions are as follows: Sex (0 = Female, 1 = Male); HBP: high blood pressure; DM: diabetes mellitus; HPL: hyperlipidemia; CHD: coronary heart disease; AF: atrial fibrillation; CVD: chronic cardiovascular disease; HT: hemorrhagic transformation; BMI: body mass index; NIHSS score: National Institutes of Health Stroke Scale score on admission; CRP: C-reactive protein; NGT: nasogastric tube. Previous stroke: values 0–3 represent the number of previous stroke times. Infarction location: 1 = anterior circulation infarction; 2 = posterior circulation infarction; 3 = combined anterior and posterior circulation infarction. Exclusive NGT feeding: complete dependence on NGT feeding at admission. Continuous variables are presented as mean (standard deviation), and categorical variables were presented as counts (percentages). The *p*^^^ represents the comparison between the training and internal validation cohorts.

### 3.2 Variable selection

**Figure 1:**
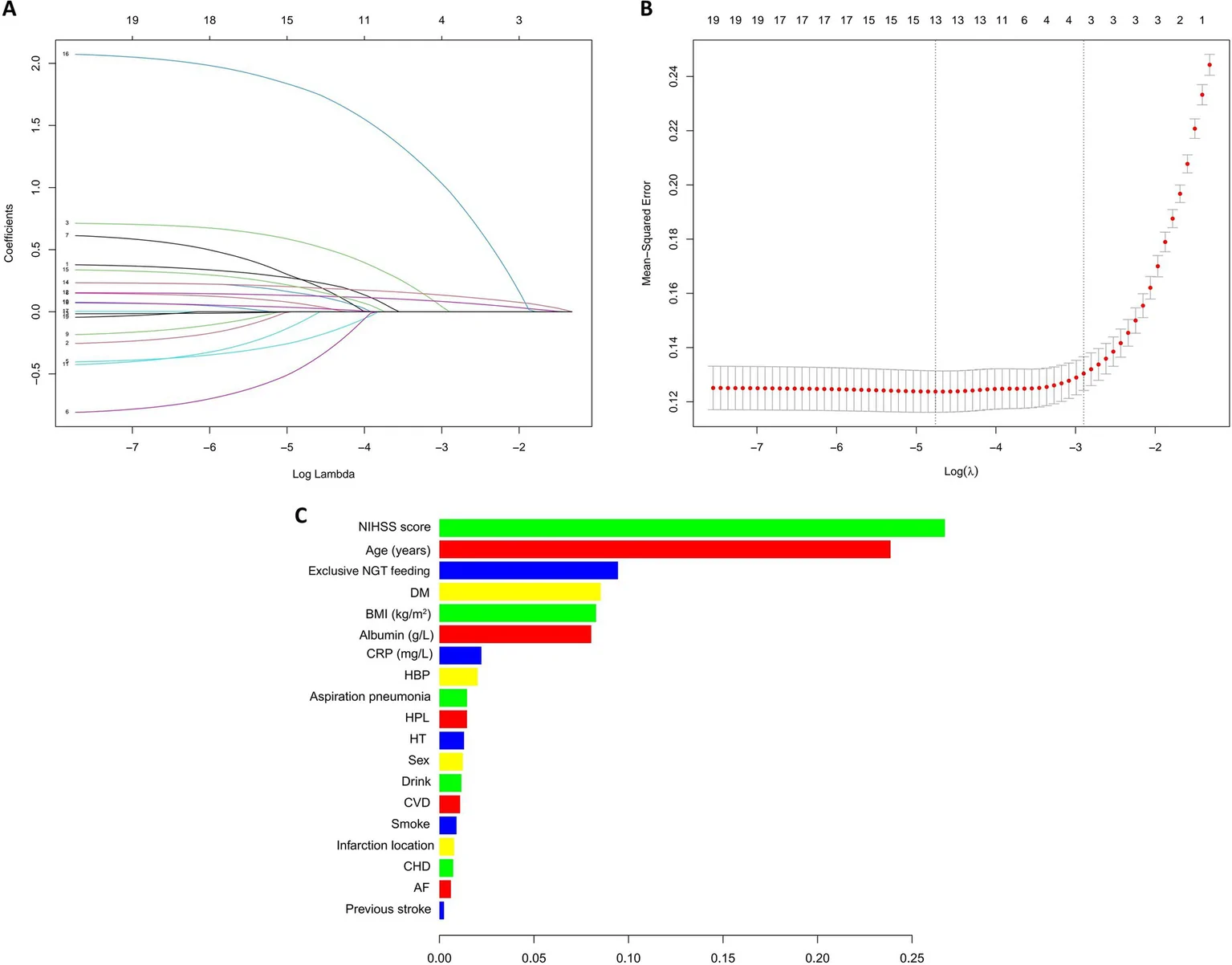
Variable selection for predicting 30-day NGT dependence. **(A)** LASSO coefficient profiles of the 19 clinical features. **(B)** The cross-validation curves of the LASSO regression analysis employ a ten-fold cross-validation approach, employing a minimum selection criterion to ascertain the optimal penalty coefficient (*λ*). **(C)** Variable importance ranking derived from the XGBoost algorithm for identifying key predictors. LASSO: least absolute shrinkage and selection operator; XGBoost: eXtreme Gradient Boosting.

LASSO regression was applied to screen 19 baseline variables and select the most relevant predictors of NGT dependence. The coefficients of most variables shrank toward zero as the regularization parameter (*λ*) increased (Figure 1A). The optimal value of λ was determined based on the minimum mean-squared error (Figure 1B), and four variables were retained for subsequent analysis: DM, age, NIHSS score, and exclusive NGT feeding.

To complement LASSO feature selection, the XGBoost algorithm was additionally applied to rank variable importance. The top-ranked predictors identified by XGBoost included NIHSS score, age, exclusive NGT feeding, and DM (Figure 1C), which were consistent with those selected by LASSO regression.

In parallel, univariate logistic regression analysis examined all 19 baseline variables to identify factors potentially associated with NGT dependence. Significant variables (*p* < 0.05) from the univariate analysis were carried forward to multivariable logistic regression, revealing five factors that independently predicted NGT dependence. The result indicated that DM (OR = 2.194, 95% CI: 1.228 to 3.975, *p* = 0.0086), AF (OR = 0.456, 95% CI: 0.21 to 0.975, *p* = 0.0445), age (OR = 1.166, 95% CI: 1.127 to 1.21, *p* < 0.001), NIHSS score (OR = 1.265, 95% CI: 1.199 to 1.341, *p* < 0.001), and exclusive NGT feeding (OR = 7.866, 95% CI: 4.592 to 13.934, *p* < 0.001) were independent predictors of NGT dependence (Table 2).

**Table 2:** Univariable and multivariable logistic regression analyses.

Variable	Univariable OR (95% CI)	*p*	Multivariable OR (95% CI)	*p*
Sex	1.467 (1.051 to 2.054)	0.0248	1.440 (0.799 to 2.622)	0.2283
HBP	1.907 (1.374 to 2.656)	<0.001	0.782 (0.435 to 1.389)	0.4052
DM	2.680 (1.858 to 3.889)	<0.001	2.194 (1.228 to 3.975)	0.0086
HPL	2.270 (1.559 to 3.322)	<0.001	1.342 (0.698 to 2.583)	0.3772
CHD	0.623 (0.406 to 0.943)	0.0272	0.641 (0.328 to 1.231)	0.1856
AF	1.700 (1.070 to 2.712)	0.0249	0.456 (0.210 to 0.975)	0.0445
CVD	2.133 (1.365 to 3.359)	0.0438	1.653 (0.757 to 3.630)	0.2079
Smoke	1.427 (1.010 to 2.018)	<0.001	1.164 (0.626 to 2.156)	0.6295
Drink	1.044 (0.658 to 1.646)	0.8532		
Previous stroke	1.037 (0.772 to 1.385)	0.8067		
HT	3.289 (2.189 to 4.997)	<0.001	0.637 (0.327 to 1.234)	0.1826
Age	1.159 (1.128 to 1.193)	<0.001	1.166 (1.127 to 1.210)	<0.001
BMI	0.977 (0.924 to 1.032)	0.4014		
NIHSS score	1.257 (1.211 to 1.309)	<0.001	1.265 (1.199 to 1.341)	<0.001
Aspiration pneumonia	1.796 (1.267 to 2.550)	0.0010	1.448 (0.840 to 2.504)	0.1831
Exclusive NGT feeding	4.377 (3.104 to 6.215)	<0.001	7.866 (4.592 to 13.934)	<0.001
CRP	1.000 (0.990 to 1.009)	0.9174		
Albumin	1.040 (0.990 to 1.092)	0.1182		
Infarction location	0.937 (0.665 to 1.313)	0.7060		

### 3.3 Construction and visualization of the prediction model

**Figure 2:**
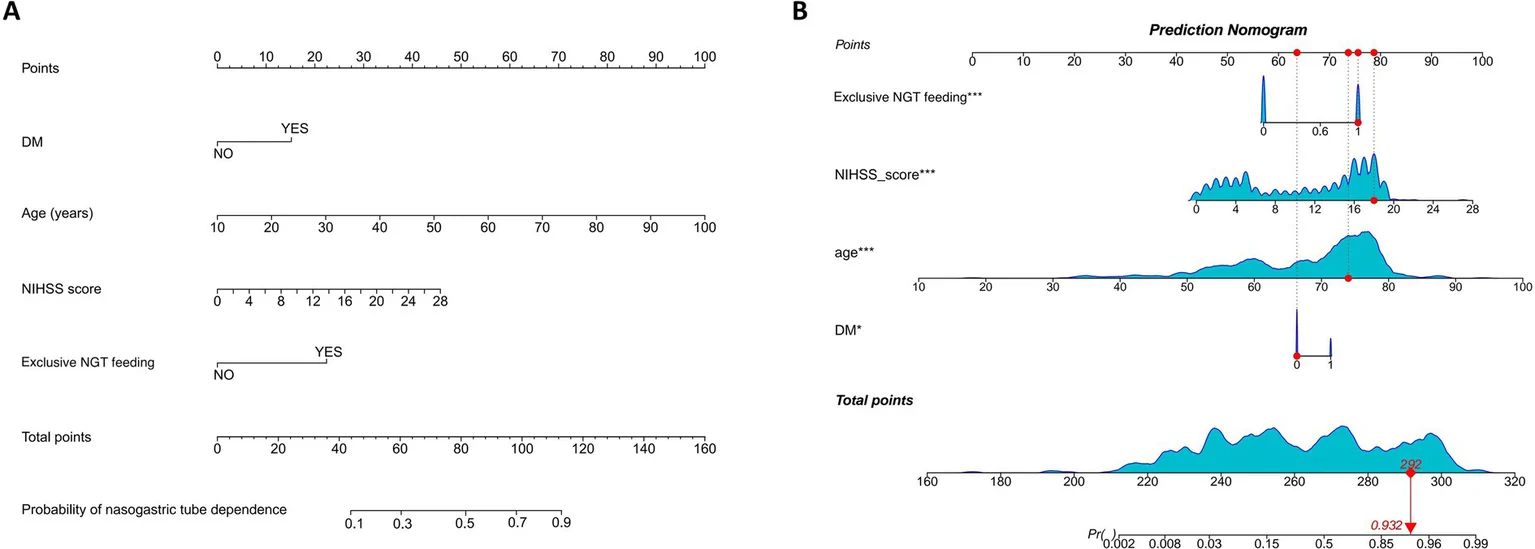
Nomogram and dynamic prediction model for 30-day NGT dependence after AIS. **(A)** Static nomogram. **(B)** Dynamic nomogram. NGT: nasogastric tube; AIS: acute ischemic stroke.

The intersection of variables identified by LASSO, XGBoost, and multivariate logistic regression analyses—DM, age, NIHSS score, and exclusive NGT feeding—was subsequently employed to construct a nomogram estimating the probability of 30-day NGT dependence in AIS patients (Figure 2A).

Illustrated the relative impact of each predictor on the risk of 30-day NGT dependence. Points were assigned to individual variables proportionally to their regression coefficients, with the total score reflecting the estimated probability of persistent NGT dependence. Among these predictors, NIHSS score contributed the highest point value, followed by exclusive NGT feeding, age and DM, indicating their relative importance in the prediction model (Figure 2B).

### 3.4 Evaluation of the nomogram’s performance

**Figure 3:**
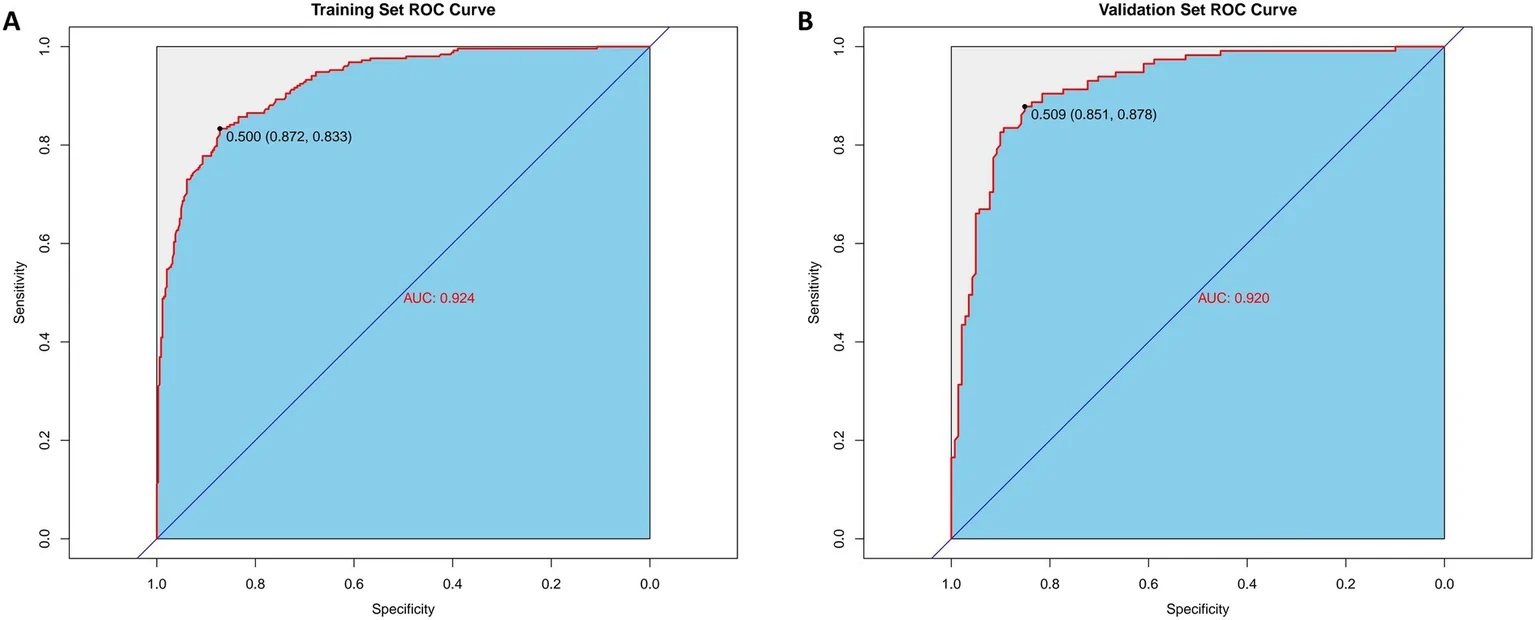
ROC curve and AUC of the nomogram model. **(A)** The ROC in the training set. **(B)** The ROC in the validation set. ROC: receiver operating characteristic; AUC: area under the curve.

The discriminative ability of the nomogram was assessed using ROC curve analysis. In the training set, the model achieved an AUC of 0.924 (95% CI: 0.904 to 0.945), with sensitivity and specificity of 0.833 and 0.872, respectively, indicating robust predictive performance (Figure 3A). In the internal validation set, the AUC was 0.920 (95% CI: 0.887 to 0.954), with corresponding sensitivity and specificity values of 0.878 and 0.851, respectively, confirming excellent predictive capability (Figure 3B).

**Figure 4:**
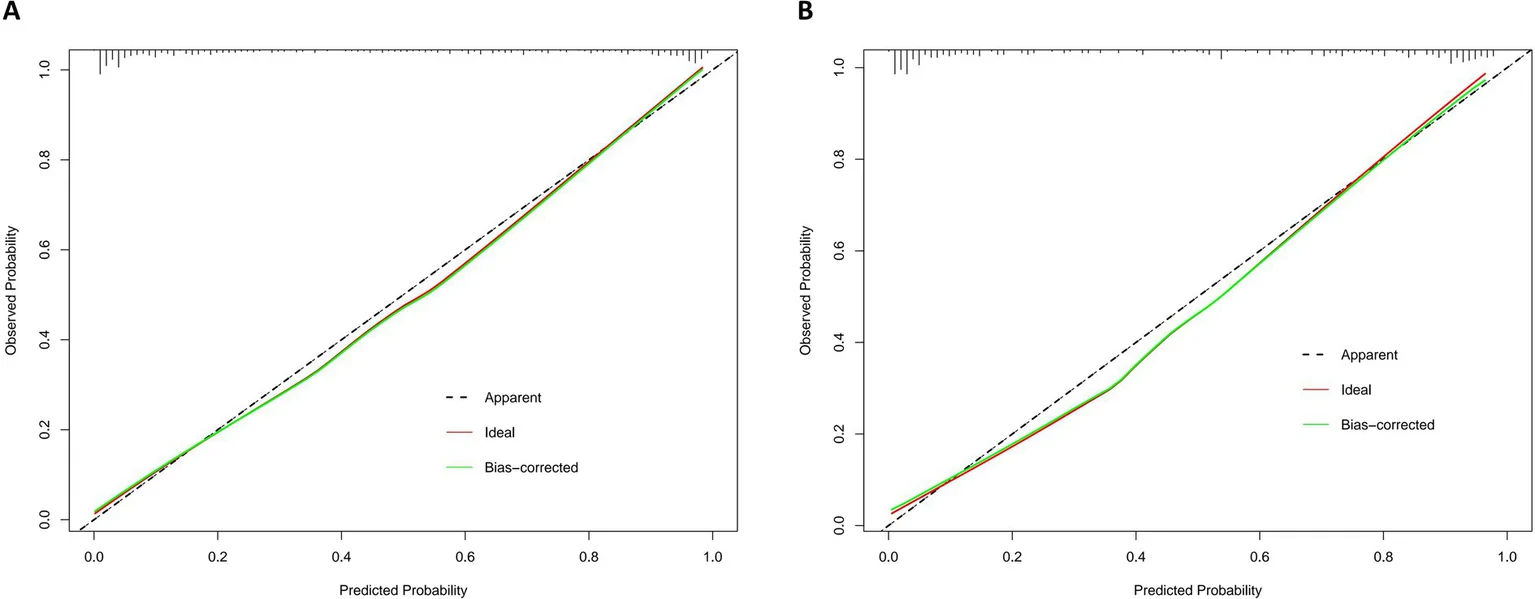
Calibration plots of the nomogram model. **(A)** Calibration plot in the training set. **(B)** Calibration plot in the validation set. The *x*-axis is the predicted probability of NGT dependence. The *y*-axis is the observed NGT dependence.

Calibration analysis indicated that the predicted probabilities of 30-day NGT dependence closely matched the observed outcomes. In both the training and internal validation sets, the bias-corrected curves closely followed the ideal 45-degree reference line, indicating excellent model calibration (Figures 4A,B).

**Figure 5:**
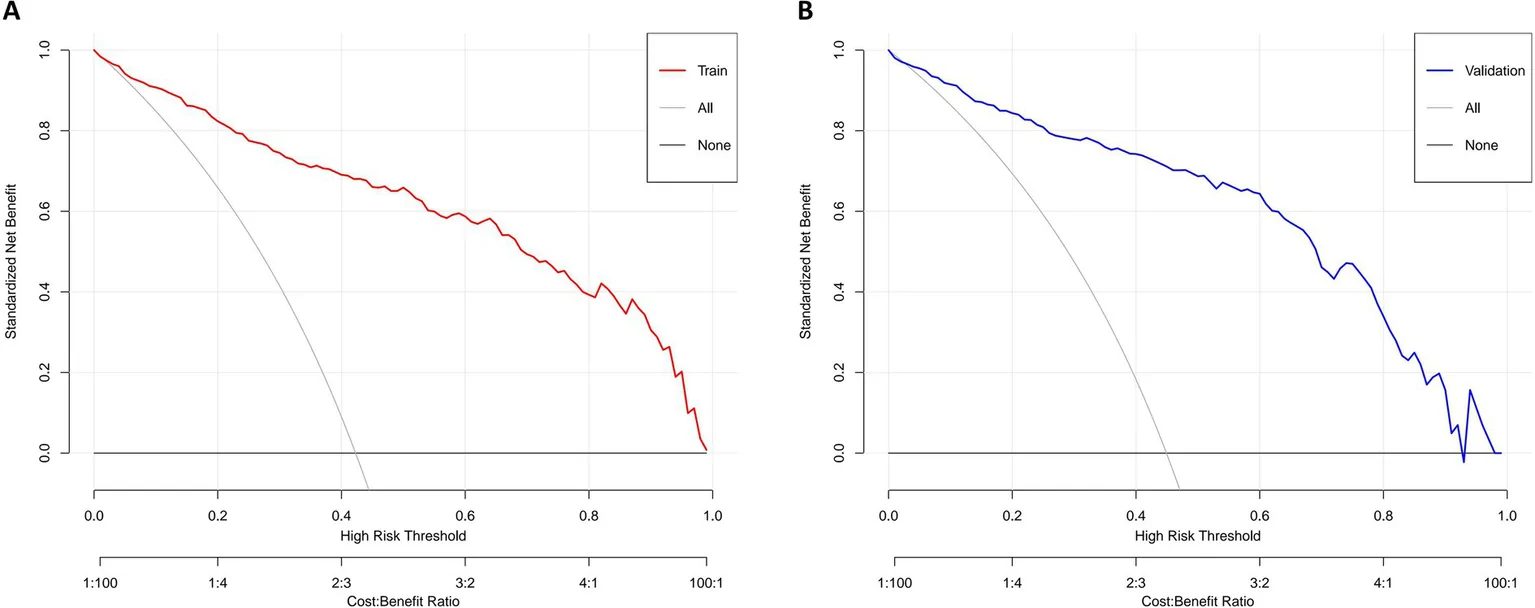
DCA for the nomogram model. **(A)** DCA for the training set. **(B)** DCA for the validation set. DCA: decision curve analysis. Black line: No patient having NGT dependence. Gray line: All patients experienced NGT dependence. Blue line: Model of nomogram.

DCA additionally validated the clinical value of the nomogram. Across a wide range of threshold probabilities, the model provided greater net benefit than both the “treat-all” and “treat-none” strategies in the training (Figure 5A) and internal validation sets (Figure 5B), indicating substantial clinical utility in predicting NGT dependence in AIS patients.

### 3.5 External validation of the nomogram model

**Figure 6:**
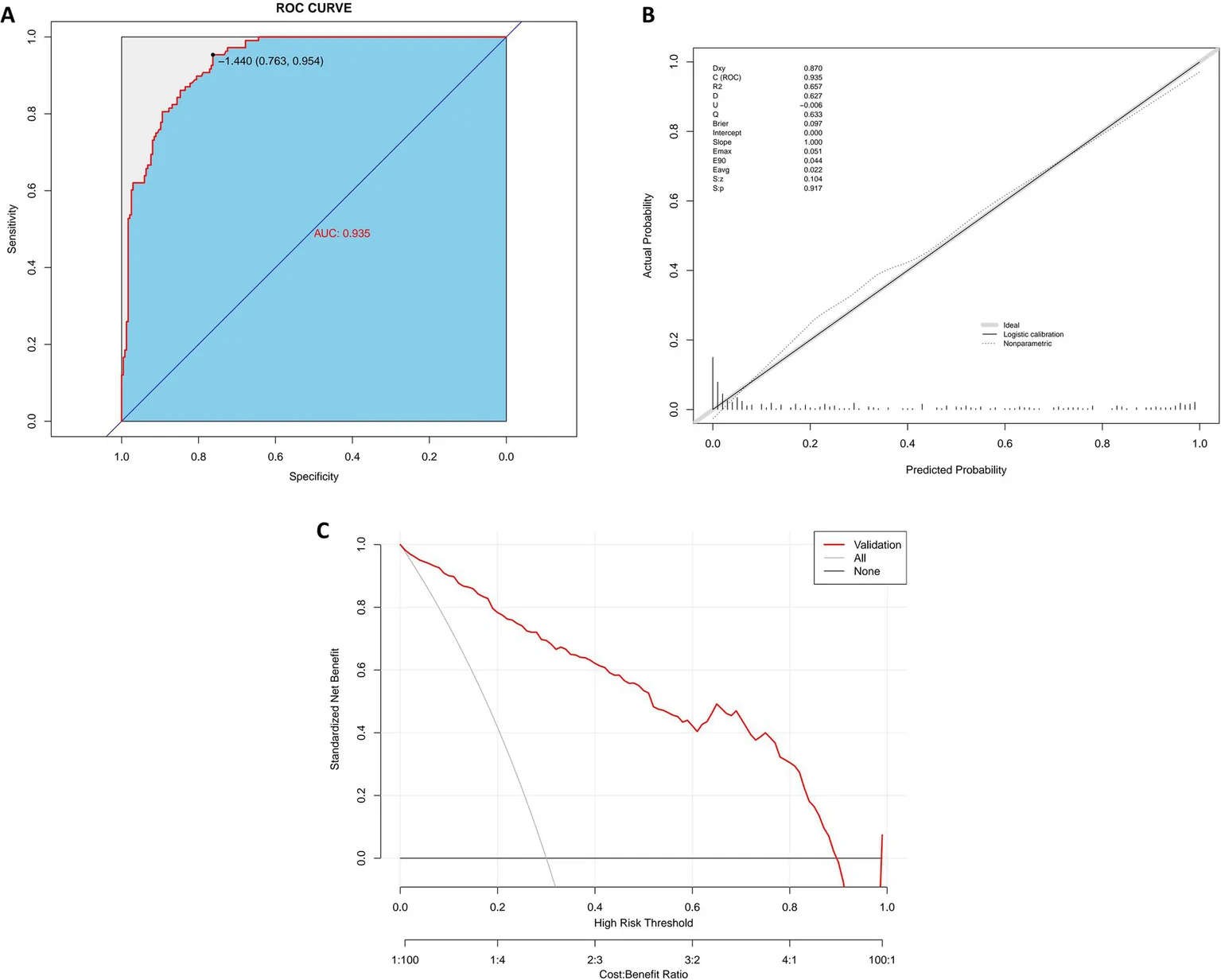
External validation of the nomogram model for discrimination, calibration, and clinical utility **(A)** ROC curve. **(B)** Calibration curve. **(C)** DCA for the external validation. ROC: receiver operating characteristic; AUC: area under the curve; DCA: decision curve analysis.

External validation was conducted using a separate, independent cohort to evaluate the generalizability of the nomogram for predicting 30-day NGT dependence in AIS patients. The nomogram exhibited strong discriminative ability, achieving an AUC of 0.935 in the external validation set, which was comparable to that of the internal validation (AUC = 0.920) (Figure 6A).

Calibration assessment revealed strong concordance between the model’s predicted probabilities and actual rates of NGT dependence. The bias-corrected calibration curve closely aligned with the ideal diagonal line, indicating excellent model calibration (Figure 6B).

DCA provided additional evidence supporting the clinical value of the model. Over a broad spectrum of threshold probabilities, the nomogram consistently delivered greater net benefit than either the “treat-all” or “treat-none” approaches, underscoring its practical utility in identifying patients at elevated risk of prolonged NGT dependence (Figure 6C).

To further evaluate the robustness of the model across different age groups, an age-stratified analysis was performed in the external validation cohort. The nomogram maintained excellent discriminative performance in both patients aged <60 years (AUC = 0.943) and those aged ≥60 years (AUC = 0.905) (Supplementary Figures S1A,B). Calibration plots also demonstrated good agreement between predicted and observed probabilities in both age subgroups (Supplementary Figures S1C,D), supporting the stability and generalizability of the model despite the different age distribution of the external validation cohort.

### 3.6 Comparison of machine learning models

To further assess the predictive robustness of the established model, three machine learning algorithms-GBM, SVM, and RDA-were developed using the same predictor set. Model performance was evaluated through five repeated resampling iterations, and the mean values of key performance metrics (AUC, Precision, Recall, and F1-score) with corresponding IQR. Among the three algorithms, the GBM model exhibited the strong discriminative ability, achieving the median AUC of 0.918 (IQR: 0.918 to 0.944). In addition, the GBM model yielded the highest Precision and F1-score values across the resampling iterations, indicating superior overall predictive accuracy and balance between sensitivity and specificity (Table 3).

**Table 3:** Comparison of model performance metrics for GBM, SVM, and RDA.

Model type	AUC Median (IQR)	Precision Median (IQR)	Recall Median (IQR)	F1-score Median (IQR)
GBM	0.918 (0.918 to 0.944)	0.896 (0.843 to 0.906)	0.870 (0.868 to 0.884)	0.872 (0.861 to 0.882)
SVM	0.906 (0.881 to 0.908)	0.862 (0.847 to 0.871)	0.884 (0.868 to 0.884)	0.865 (0.855 to 0.878)
RDA	0.931 (0.917 to 0.933)	0.871 (0.859 to 0.887)	0.826 (0.797 to 0.870)	0.863 (0.840 to 0.870)

Values are presented as median (interquartile range [IQR], 1st-3rd quartile) based on five resampling iterations. AUC indicates the area under the ROC curve; Precision reflects the proportion of correctly identified positive predictions; Recall (sensitivity) represents the proportion of true positives identified; and F1-score is the harmonic mean of Precision and Recall.

**Figure 7:**
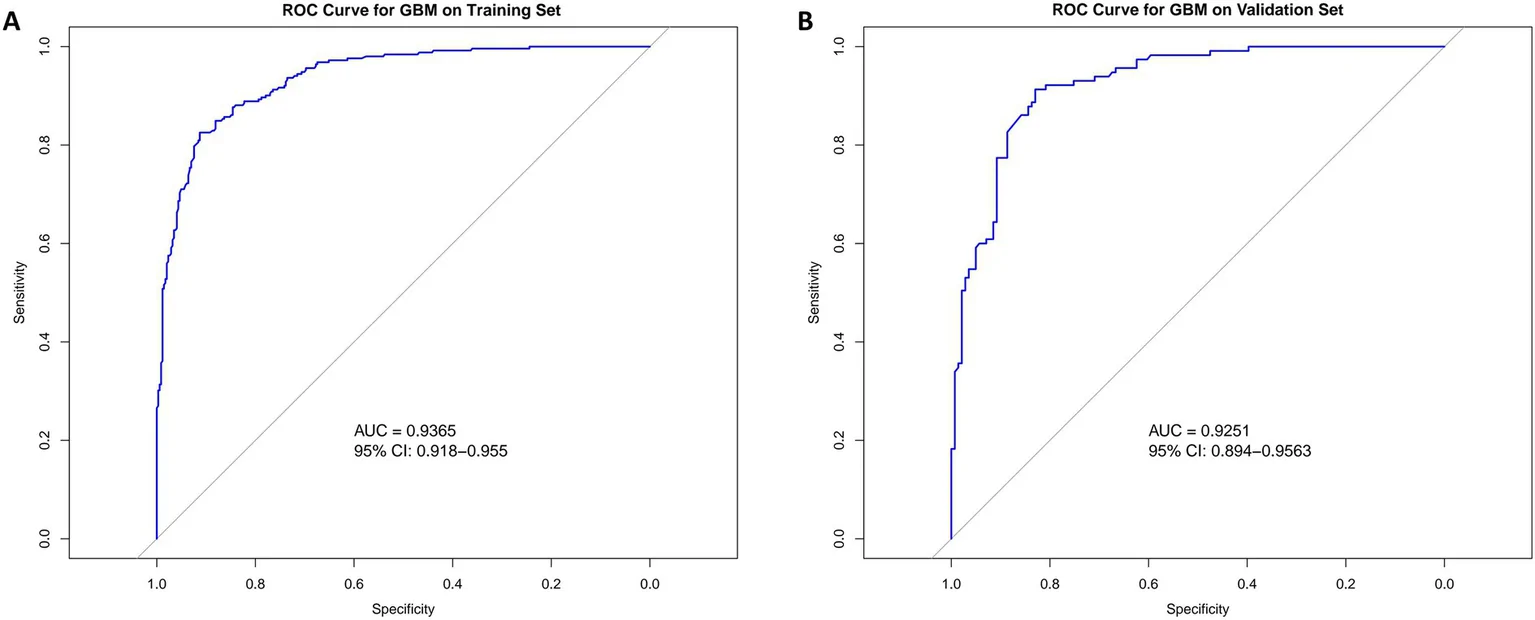
ROC curves of the GBM model. **(A)** Training set. **(B)** Validation set. GBM: gradient boosting machine; ROC: receiver operating characteristic; AUC: area under the curve.

**Figure 8:**
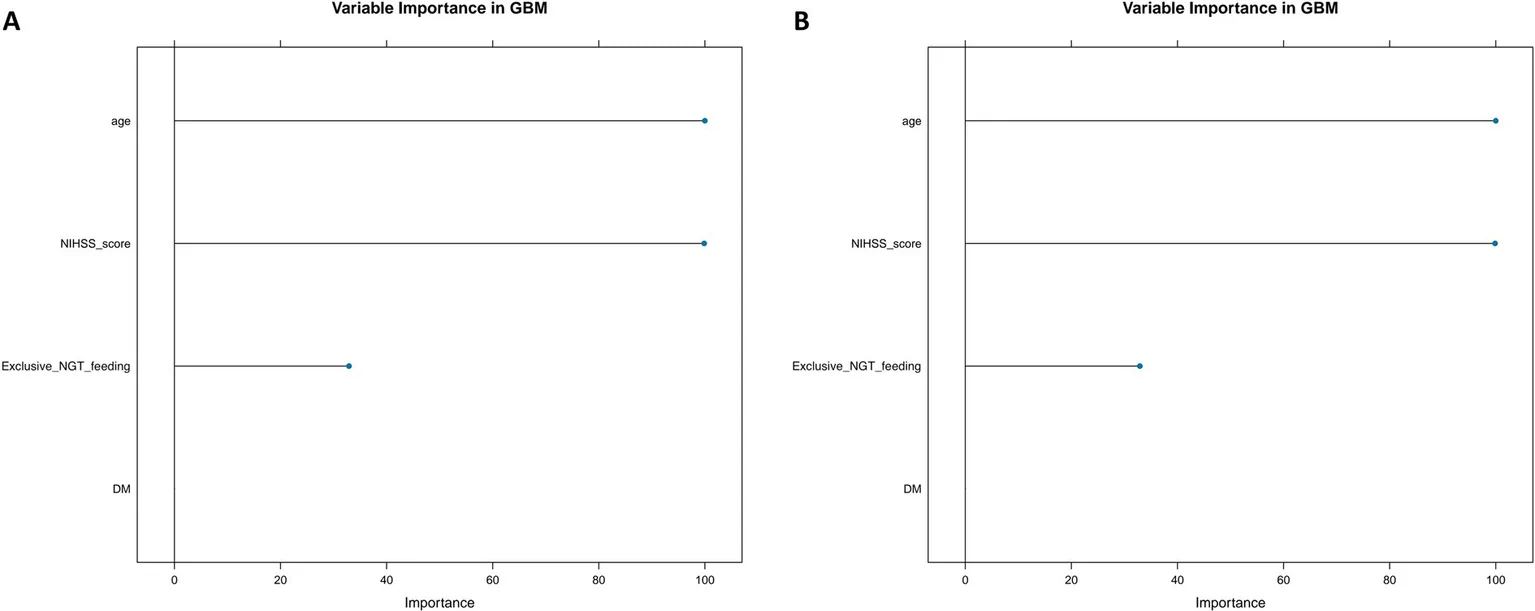
Relative importance of variables in the GBM model. **(A)** Training set. **(B)** Validation set. GBM: gradient boosting machine.

The ROC curves for the GBM model in both the training and internal validation cohorts further confirmed its robust predictive performance, with AUCs of 0.937 (95%CI: 0.918 to 0.955) in training set (Figure 7A) and 0.925 (95% CI: 0.894 to 0.956) in internal validation set (Figure 7B). The lollipop plot visualized the relative importance of the predictors within the GBM model, identifying age, NIHSS score, and exclusive NGT feeding as the most influential variables associated with NGT dependence in both training and internal validation sets (Figure 8).

## 4 Discussion

A predictive model for 30-day NGT dependence in AIS patients was developed and validated using integrated statistical and machine learning approaches. By integrating the strengths of multivariate logistic regression, LASSO regression, and the XGBoost algorithm, four independent predictors-DM, age, NIHSS score, and exclusive NGT feeding-were identified and incorporated into a nomogram. The model demonstrated excellent predictive performance and calibration in both the training and internal validation cohorts, which was further supported by external validation. Furthermore, comparison among three machine learning algorithms revealed that the GBM model demonstrated the highest predictive accuracy, supporting the reliability of the selected predictors in forecasting 30-day NGT dependence.

Dysphagia affect more than half of 800,000 people who experience a stroke annually in the United States (29). In the U. S., the annual cost of hospital care and rehabilitation for dysphagia patients exceeds $500 million, imposing a substantial economic burden on healthcare systems (30). Post-stroke dysphagia contributes to aspiration pneumonia, dehydration, and prolonged hospitalization, resulting in higher medical costs and poorer outcomes (31). Additionally, dysphagia independently increases the risk of malnutrition, impaired functional recovery, and post-stroke depression (6). To minimize these adverse outcomes, clinicians must decide within the first 48 h if enteral tube feeding is necessary (32). Furthermore, the restoration of safe oral intake has emerged as a critical determinant of hospital discharge for stroke patients (33).

NGT feeding is typically intended as a short-term intervention while awaiting improvement in swallowing function. Clinical guidelines recommend maintaining NGT feeding when oral intake remains inadequate for at least one week (12, 34). However, if dysphagia persists beyond approximately four weeks, patients are generally considered candidates for long-term enteral access, such as percutaneous endoscopic gastrostomy or jejunostomy (35). Prior research has reported that the rate of successful NGT removal ranges from 31 to 74% (18, 22, 36), indicating variability in recovery trajectories. Persistent NGT dependence after AIS reflects delayed recovery of swallowing function. Previous studies reported multiple factors linked to persistent dysphagia after stroke, including older age (37), delayed swallowing rehabilitation (38), bilateral cerebral infarctions (19), higher admission NIHSS scores (39), and facial palsy or aphasia (40, 41). These findings suggest that both neurological severity and impaired neuromuscular coordination contribute to long-term feeding dependence.

Among the predictors retained in our final model, age and NIHSS score emerged as the most influential predictors of 30-day NGT dependence in GBM analyses, which was aligned with prior literature (18–20). Research indicates that younger stroke patients are significantly more likely to achieve NGT removal before hospital discharge than the elderly, with age serving as a predictor for oral feeding outcomes (42, 43). Age-related atrophy of swallowing musculature, including reduced thickness of the tongue, geniohyoid muscle, and pharyngeal wall, along with enlargement of the pharyngeal lumen, can impair swallowing efficiency. These structural changes are associated with delayed pharyngeal trigger, prolonged oral–pharyngeal transit, reduced pharyngeal contractility, and diminished endurance of the swallowing muscles (44, 45). Thus, age-related muscle atrophy could influence the dysphagia outcome.

The NIHSS score is used to determine stroke severity, monitor changes over time, and guide treatment decisions (46). Higher NIHSS scores indicate more extensive neurological impairment, particularly in cortical and brainstem regions governing swallowing and airway defense, thereby elevating the risk of persistent dysphagia (47). In patients with anterior large-vessel occlusion treated with EVT, higher NIHSS scores correlated strongly with post-stroke dysphagia (23). Similarly, among 310 patients with intracerebral hemorrhage, NIHSS score at 24 h was a significant predictor of continued NGT insertion at 4 weeks (48). Another study reported that 22% of patients remained NGT-dependent after 4 weeks of rehabilitation, with age, improvement in motor function, and NIHSS item 9 serving as independent predictors of successful NGT removal (11).

Notably, our study also identified DM and exclusive NGT feeding status as significant contributors to persistent feeding dependence. Diabetes has been linked to autonomic neuropathy and microvascular injury, which may disrupt neuromuscular control of swallowing and delay functional recovery (49). However, although DM is a recognized risk factor for stroke, prior studies did not find a significant relationship between DM and NGT removal in patients with post-stroke dysphagia (11, 22). NGT feeding delivers nutrition through a narrow tube inserted via the nostril, passed along the nasal passage, and advanced into the stomach (50). Unlike partial dependence, which allows patients to tolerate some oral intake, total dependence reflects more severe impairment of swallowing physiology at baseline, serving as a practical clinical marker for early risk stratification (7, 51). As a readily available clinical indicator assessed at admission, it may help identify patients at increased risk of persistent NGT dependence and facilitate individualized swallowing rehabilitation, nutritional management, and timely consideration of long-term enteral feeding strategies.

Although our model incorporated readily available clinical predictors, other factors may also contribute to persistent NGT dependence after AIS. Emerging evidence suggests that chronic cerebral small vessel disease, particularly silent white matter alterations, is associated with cognitive impairment and may adversely affect functional recovery during aging (52, 53). These structural abnormalities may provide complementary prognostic information beyond conventional clinical variables. However, because neuroimaging markers such as white matter hyperintensity burden or Fazekas score were not routinely available in our retrospective cohort, their predictive value could not be evaluated. Future studies integrating structural neuroimaging with clinical variables may further improve risk stratification for persistent NGT dependence.

Future studies should also explore additional determinants of persistent NGT dependence that were beyond the scope of the present study. In particular, standardized ischemic stroke subtype classification deserves further investigation, as different stroke subtypes differ substantially in vascular risk factor profiles, stroke severity, treatment strategies, and functional outcomes, which may also influence swallowing recovery and long-term enteral feeding requirements (54). Furthermore, prospective multicenter studies with larger sample sizes, longitudinal swallowing assessments, and dynamic evaluation of rehabilitation interventions are warranted to validate and optimize prediction models across diverse stroke populations. Such efforts may facilitate more individualized nutritional support and swallowing rehabilitation strategies in clinical practice.

## 5 Limitations

Several limitations should be noted. First, this was a single-center study, which may restrict the applicability of the findings to other institutions due to variations in clinical practices and patient selection. Second, several potentially relevant predictors were not incorporated into the model. These included neuroimaging markers of chronic cerebral small vessel disease, such as white matter hyperintensities and other silent vascular lesions, as well as lesion volume, imaging-confirmed brainstem involvement, standardized ischemic stroke subtype classification, detailed nutritional parameters, and rehabilitation intensity. The absence of these variables may have limited the predictive performance of the model, and their incremental value should be explored in future prospective studies. Third, although the model demonstrated consistent performance across age subgroups in the external validation cohort, the relatively small sample size and younger age distribution of this cohort may have limited the precision of subgroup estimates. Validation in larger and more age-diverse external cohorts is warranted to further confirm the robustness and generalizability of the model. Finally, the definition of persistent dysphagia was based solely on the WST score, which does not capture the full spectrum of swallowing physiology and may underestimate silent aspiration. Further large-scale, prospective, multicenter studies incorporating multimodal predictors and extended follow-up are required to validate and enhance the clinical utility of this predictive model.

## 6 Conclusion

In summary, this study established and validated a robust nomogram and machine learning models for predicting 30-day NGT dependence in AIS patients. The integration of demographic, metabolic, and neurological indicators enabled high-accuracy risk stratification, with age, NIHSS score, exclusive NGT feeding, and DM emerging as the most influential predictors. The proposed model offers a clinically practical tool to support individualized NGT removal decision-making, potentially improving patient outcomes and optimizing intensive care resource utilization. Large-scale, multicenter, prospective research is needed to externally assess and refine the model for routine clinical use.

## Data Availability

The original contributions presented in the study are included in the article/Supplementary material, further inquiries can be directed to the corresponding author.

## Supplementary material

SUPPLEMENTARY FIGURE 1 Age-stratified validation of the nomogram in the external validation cohort. **(A)** ROC curve for patients aged <60 years. **(B)** ROC curve for patients aged ≥60 years. **(C)** Calibration curve for patients aged <60 years. **(D)** Calibration curve for patients aged ≥60 years.

## Glossary

AIS: Acute ischemic stroke
AF: Atrial fibrillation
AUC: Area Under the Curve
BMI: Body mass index
CHD: Coronary heart disease
CI: Confidence interval
CRP: Serum C-reactive protein
CT: Computed tomography
CVD: Chronic cardiovascular disease
DCA: Decision Curve Analysis
DM: Diabetes Mellitus
GBM: Gradient Boosting Machine
HBP: High blood pressure
HPL: Hyperlipidemia
HT: Hemorrhagic transformation
IQR: Interquartile Range
LASSO: Least absolute shrinkage and selection operator
MRI: Magnetic resonance imaging
NGT: Nasogastric tube
NIHSS: National Institutes of Health Stroke Scale
OR: Odds ratio
RDA: Regularized Discriminant Analysis
ROC: Receiver operating characteristic
SVM: Support Vector Machine
WST: Water Swallow Test
XGBoost: eXtreme Gradient Boosting
